# LONGITUDINAL RELATIONSHIP BETWEEN ENERGY UTILIZATION AND PHYSICAL AND COGNITIVE PERFORMANCE AS A FUNCTION OF AGE

**DOI:** 10.1093/geroni/igz038.1795

**Published:** 2019-11-08

**Authors:** Pei-Lun Kuo, Yang An, Alden Gross, Eleanor M Simonsick, Susan M Resnick, Jennifer A Schrack

**Affiliations:** 1 Johns Hopkins University Bloomberg School of Public Health, Baltimore, Maryland, United States; 2 Laboratory Of Behavioral Neuroscience, National Institute On Aging, NIH, Baltimore, Maryland, United States; 3 Longitudinal Studies Section, Translational Gerontology Branch, National Institute On Aging, NIH, Baltimore, Maryland, United States; 4 Johns Hopkins University, Baltimore, Maryland, United States

## Abstract

Energy utilization, which becomes more inefficient with age and is measured by a ratio of energy-cost-to-energy-capacity (“cost-ratio”), has been associated with functional decline. However, the interplay between longitudinal changes in energy efficiency and physical/cognitive functioning remains unclear. We investigated this relationship in 1020 participants of the Baltimore Longitudinal Study of Aging (baseline age: 68.9 (IQR: 59.8, 80.5), male: 44.7%). In linear mixed effects models adjusted for baseline age, sex, and height, an increasing cost-ratio was associated with faster decline in usual gait speed among those aged 50-64 years (beta = -0.20 m/s, p = 0.003), and >=65 years (beta = -0.16 m/s, p less than 0.001), but not those less than 50 years (beta = -0.22 m/s, p = 0.178). In models adjusted for baseline age, sex, race, and years of education, higher cost-ratio was associated with faster declines in executive function, as measured by time to finish Trail B, among those aged >=65 years (beta = 22.96 seconds, p = 0.020), but not <50 years (beta = -13.65 seconds, p = 0.557) or 50-64 years (beta = -15.61 seconds, p = 0.353). Together, these results suggest that energy utilization becomes more inefficient in the two to three decades prior to change in physical and cognitive functioning, implying it may act as an early marker of physiological aging. Further research is needed to understand the drivers of energy inefficiency, which may shed light on the biological mechanisms contributing to these declines.

